# Phytophagy of omnivorous predator *Macrolophus pygmaeus* affects performance of herbivores through induced plant defences

**DOI:** 10.1007/s00442-017-4000-7

**Published:** 2017-11-09

**Authors:** Nina Xiaoning Zhang, Gerben J. Messelink, Juan M. Alba, Robert. C. Schuurink, Merijn R. Kant, Arne Janssen

**Affiliations:** 1 IBED, Population Biology, University of Amsterdam, Science Park 904, 1098 XH Amsterdam, The Netherlands; 2 Wageningen UR Greenhouse Horticulture, PO Box 20, 2265 ZG Bleiswijk, The Netherlands; 3 Department of Plant Physiology, Swammerdam Institute for Life Sciences, University of Amsterdam, P.O. Box 94215, 1090 GE Amsterdam, The Netherlands

**Keywords:** *Myzus persicae*, *Tetranychus urticae*, *Frankliniella occidentalis*, Plant hormones

## Abstract

Plants possess various inducible defences that result in synthesis of specialized metabolites in response to herbivory, which can interfere with the performance of herbivores of the same and other species. Much less is known of the effects of plant feeding by omnivores. We found that previous feeding of the omnivorous predator *Macrolophus pygmaeus* on sweet pepper plants significantly reduced reproduction of the two-spotted spider mite *Tetranychus urticae* and western flower thrips *Frankliniella occidentalis* on the same plants, also on leaves that had not been exposed to the omnivore. In contrast, no effect was found on the reproduction of the green peach aphid *Myzus persicae*. Juvenile survival and developmental time of *T. urticae* and *M. persicae,* and larval survival of *F. occidentalis* were not affected by plant feeding by *M. pygmaeus*. Larvae of *F. occidentalis* feeding on leaves previously exposed to *M. pygmaeus* required longer to develop into adults. Defence-related plant hormones were produced locally and systemically after exposure to *M. pygmaeus*. The concentrations of 12-oxo-phytodienoic acid and jasmonic acid–isoleucine in the attacked leaves were significantly higher than in the corresponding leaves on the uninfested plants, and jasmonic acid concentrations showed the same trend, suggesting that jasmonic-acid-related defence pathways were activated. In contrast, similar concentrations of salicylic acid were found in the attacked leaves of *M. pygmaeus*-infested plants and uninfested plants. Our results show that plant feeding by omnivorous predators decreases the performance of herbivores, suggesting that it induces plant defences.

**Electronic supplementary material:**

The online version of this article (10.1007/s00442-017-4000-7) contains supplementary material, which is available to authorized users.

## Introduction

Plants employ different types of defences to resist herbivores. Such defences can be displayed constitutively or can be induced. In general, constitutive defences are present all the time, and induced plant defences are reinforced or activated by herbivory, involving the production of signalling molecules, which results in the upregulation of biosynthesis of specific compounds such as toxins and digestion inhibitors (Karban and Baldwin 1997; Kant et al. 2015). These compounds act directly against herbivores, reducing their growth or survival or reproductive rate (Howe and Jander 2008; Kant et al. 2015). Plants can also defend themselves indirectly by involving the natural enemies of the herbivores (Price et al. 1980), for example, by providing them with food or shelter, or by attracting them with volatiles induced by herbivore damage (Sabelis et al. 1999a, b). Induced plant defences can be local, i.e. expressed at the damaged site, and systemic, i.e. expressed in plant parts that were not damaged by the herbivores. At the damaged sites of the plant, signals may be produced and transmitted systemically to undamaged distal sites, resulting in priming or induction of plant defences in those sites, which provides resistance to future herbivore attacks (Karban and Baldwin 1997; Howe and Jander 2008; Heil and Ton 2008; Conrath et al. 2015). Phytohormones play important roles in regulating induced defences (Pieterse et al. 2009). The most important hormones involved in induced defences are jasmonic acid (JA), salicylic acid (SA) and the hormone ethylene (Erb et al. 2012; Pieterse et al. 2012).

Plant defences can differ qualitatively and quantitatively with the herbivore species attacking the plants (de Vos et al. 2005; Rodriguez-Saona et al. 2010) and time since attack (Kant et al. 2004). Moreover, different defence pathways can affect each other either positively or negatively (Koornneef and Pieterse 2008). Therefore, herbivores sharing the same plant can affect each other through the defences they induce (Rodriguez-Saona et al. 2005; Kaplan et al. 2009). Much research has been conducted into such indirect herbivore interactions mediated via induced plant defences, but little information is available on the induction of plant defences by omnivorous predators and their effects on herbivore performance on the same plant. Omnivorous predators are important in natural ecosystems (Coll and Guershon 2002) and are increasingly used as biological control agents because they can feed on plant material and their populations can thus persist in the crop when pest densities are low, making the system more resilient (Messelink et al. 2012). However, some omnivores can also damage the plants (Puentes and Björkman 2017), hence these omnivores are less useful for biological control.

Plant feeding by some omnivores is known to activate plant defence mechanisms. For example, the predatory bug *Orius laevigatus* was shown to increase resistance of tomato plants against thrips and whiteflies (De Puysseleyr et al. 2011). In addition, Pérez-Hedo et al. (2015b) and Naselli et al. (2016) have shown that exposing tomato plants to the mirid bug *Nesidiocoris tenuis* resulted in the activation of the abscisic acid (ABA) and jasmonic acid (JA) signalling pathways involved in plant defences, but not the SA signalling pathway, whereas *Macrolophus pygmaeus* activated only the JA pathway and not the ABA pathway (Pérez-Hedo et al. 2015a). Pappas et al. (2015) found that exposing tomato plants to the omnivorous predator *Macrolophus pygmaeus* reduced the performance of a subsequently infesting herbivore, the two-spotted spider mite *Tetranychus urticae*, but not of the greenhouse whitefly *Trialeurodes vaporariorum*. Here, we further investigated the effects of plant feeding by *M. pygmaeus* on herbivore performance and plant hormone levels in another plant species, sweet pepper (*Capsicum annuum* L.). We specifically tested whether this effect was local or systemic.

*Macrolophus pygmaeus* is an important omnivorous predator of several agricultural pests such as whiteflies (Montserrat et al. 2000), thrips (Riudavets and Castañé 1998), aphids (Alvarado et al. 1997), spider mites (Hansen et al. 1999), leaf miners (Arnó et al. 2003) and Lepidoptera species, including *Tuta absoluta* (Urbaneja et al. 2009). It attacks a wide range of arthropod pests and is commercially used for the biological control of spider mites and whiteflies. However, it can also feed on plant tissue (Perdikis and Lykouressis 2000). Plant damage has been observed with high predator densities on tomato, zucchini, and gerbera under experimental conditions, but no such damage has been observed under cropping conditions (Castañé et al. 2011). In this paper, we investigated the effects of phytophagy of *M. pygmaeus* on the performance of three species of its herbivorous prey, the two-spotted spider mite *Tetranychus urticae* Koch (Acari, Tetranychidae), the green peach aphid *Myzus persicae* (Hemiptera, Aphididae), and the western flower thrips *Frankliniella occidentalis* (Pergande) (Thysanoptera, Thripidae).

These herbivores species employ different feeding strategies. Spider mites and thrips are cell-content feeders, but spider mites have relatively long stylets and feed on parenchymal cells and avoid damaging the epidermis cells (Bensoussan et al. 2016), and thrips use their mouthparts to punch holes in both the epidermal cells and mesophyll cells and subsequently empty the punctured cells with their stylets, resulting in strong plasmolysis and collapse of cells (Hunter and Ullman 1992; van de Wetering et al. 1998). Aphids use their long flexible stylets to feed only from phloem, causing hardly any damage to mesophyll tissue (Walling 2008; Schwarzkopf et al. 2013). These different feeding strategies are reflected in the differential defence responses they induce in plants. Spider mites induce both the JA and the SA pathway (Kant et al. 2004), thrips predominantly induce the JA pathway (Kawazu et al. 2012), whereas phloem-feeding insects such as whiteflies and aphids generally activate the SA pathway (Walling 2000). JA-related plant defences decrease the performance of spider mites and thrips (Omer et al. 2001; Kant et al. 2008). Aphids and whiteflies are also sensitive to the JA-related defences, but they mainly induce SA-related defences, which can suppress JA-related defences (Omer et al. 2001; Zarate et al. 2007; Walling 2008; Puthoff et al. 2010). Owing to these different sensitivities to plant defences, we expected that the herbivores would be differentially affected by plant feeding by the omnivore, and this would help to evaluate which types of defences are induced by the omnivore. We therefore tested the performance of herbivores on plants previously infested by *M. pygmaeus* and on uninfested plants. To test the induction of different defensive systems by the omnivore, we quantified defence-related plant hormones of both defensive pathways in plants infested by *M. pygmaeus* and in uninfested plants. Specifically, we quantified concentrations of the two main hormones of the two defensive pathways, JA and SA. We furthermore quantified the hormone12-oxo-phytodienoic acid (OPDA), which is the precursor of JA (Wasternack and Hause 2013), JA-isoleucine (JA-Ile), which is the main bioactive form of JA and plays a key role in regulating defence gene expression (Fonseca et al. 2009), and abscisic acid (ABA) (Bodenhausen and Reymond 2007; Pieterse et al. 2009), which plays an important ancillary role in fine-tuning plant defences (Kessler and Baldwin 2002; Vos et al. 2013; Kant et al. 2015).

## Materials and methods

### Plant material

Sweet pepper plants (*Capsicum annuum* L. Spider F1 Enza Zaden Beheer B.V., The Netherlands) were grown from seeds in pots (Ø = 14 cm) with soil in a climate room dedicated to growing uninfested plants (25 ± 1 °C, 60–70% RH, 16:8 L:D). Water was supplied twice a week. Four-week-old plants with 6–8 true leaves (about 20 cm high) were used for experiments. Plants of 5–8 weeks old were used for the rearing of spider mites, thrips and aphids.

### Cultures

A culture of *M. pygmaeus* was established with fifth instar nymphs obtained from a commercial biological control company (Koppert Biological Systems BV, Berkel en Rodenrijs, The Netherlands). It was reared in plastic containers (height = 45 cm, Ø = 35 cm) in a climate room (conditions as above) with *Ephestia kuehniella* eggs as food and green bean pods, which served as both food supply and oviposition substrate. New *E. kuehniella* eggs and beans were added twice a week. Old beans with *M. pygmaeus* eggs were transferred to new containers and kept until the eggs hatched, whereupon *E. kuehniella* eggs and beans were supplied twice a week. Beans were removed from the containers when no new nymphs hatched from them. Adults of 4–8 days old were used for experiments.

A culture of *T. urticae* was started with individuals that were obtained from a cucumber culture in our lab (see Janssen 1999 for details), and reared on intact sweet pepper plants in a climate room (conditions as above). New plants were provided twice a week. The mites were cultivated for 10 months on sweet pepper plants before being used for experiments. Thus, *T. urticae* females used in the experiments were adapted to sweet pepper plants. Nevertheless, the oviposition rate of these spider mites remained low.

A red phenotype of *M. persicae* was obtained from a culture on sweet pepper plants at Wageningen UR Greenhouse Horticulture (Bleiswijk, The Netherlands). The culture was established by placing all individuals on intact sweet pepper plants in insect-proof cages (BugDorm-44545F, 47.5 × 47.5 × 47.5 cm, mesh size: 160 µm) in a climate chamber (conditions as above). New plants were supplied every 2 weeks.

A culture of *F. occidentalis* originated from the stock culture of Koppert Biological Systems and was maintained on bean pods and cattail pollen (*Typha latifolia* L.) These thrips were subsequently reared on sweet pepper plants supplemented with cattail pollen in fine mesh cages (as above) in a climate chamber (conditions as above). New pollen was applied on sweet pepper leaves with a fine brush three times per week. New plants were supplied twice per week. To obtain cohorts of thrips larvae, adult thrips were collected with an aspirator connected to a vacuum pump and placed on uninfested sweet pepper leaves on soaked cotton wool in Petri-dishes sealed with Parafilm. The lids had ventilation holes covered with a fine mesh for ventilation. Adults were removed after 24 h and leaves were kept until new larvae hatched. Thereafter, new leaves and pollen were added. In this way, cohorts of similar-aged adults were obtained for experiments.

### Infestation of sweet pepper plants with *M. pygmaeus*

Four-week-old plants with 6–8 true leaves were transferred to a climate room (conditions as above). The fourth leaves from below were treated with omnivorous predators to observe the systemic effects on untreated leaves above and below the treated leaves. To restrict the feeding of the omnivores, bags made of fine mesh (30 µm, size: 15 × 13 cm) were used to enclose them on the leaf to be treated. Five adult males and five adult females of *M. pygmaeus*, starved for 2 h, were released in a bag of half the plants, haphazardly chosen. The other half of the plants also received bags over the fourth leaf, but no omnivores, and served as controls. All bags were closed with stretchable ropes around the stems of the leaves to prevent *M. pygmaeus* from escaping. After 4 days, all adults and bags were removed from all plants. No food or prey was supplied for *M. pygmaeus* during these 4 days, thus preventing the females from ovipositing (Perdikis and Lykouressis 2004). An average of 60% of the females of *M. pygmaeus* was alive 4 days later.

### Effect of plant infestation by *M. pygmaeus* on herbivore reproduction

The second to sixth leaf (from below) of sweet pepper plants treated as above (uninfested or previously exposed to *M. pygmaeus*) were used to measure the performance of spider mites, thrips and aphids. Lanolin (Sigma-Aldrich) was pasted around the stem of each leaf of each plant to prevent spider mites from escaping. For reproduction of the spider mites, adult female *T. urticae* were collected from the culture. To reduce the effect of the previous diet on reproduction, all females were starved for 2 h before being used for the experiment. Ten starved females were carefully introduced on each leaf with a fine brush. Subsequently, the number of females was recorded on a daily basis for 4 days, and the number of eggs was recorded on the 4th day under a stereomicroscope. The experiments were conducted in 3 blocks in time and there were 5 plants of each treatment per block. We calculated the number of eggs per mite-day as follows: First, we observed that almost all mites disappeared from the 2nd leaf from below of all plants, suggesting that the quality of this leaf was low, independent of treatment. Because we could not rule out the possibility that the mites escaped by dropping from the plant immediately after being introduced, we excluded oviposition on this leaf for further analysis. Second, we summed the number of alive, ovipositing females on the other leaves per day over a 4-day period, yielding the total number of mite-days per leaf. Subsequently, the total number of eggs produced during 4 days was divided by this total number of mite-days, thus correcting for mortality or escapes of adult females. Each plant was considered as a replicate but because we were interested in local and systemic effects of the feeding by *M. pygmaeus*, we averaged oviposition per leaf per plant. These oviposition rates on different leaves of each treatment were compared using linear mixed-effects models (LME), with treatment, leaf and their interaction as fixed factors and individual plant and block as random factors. The distribution of the residuals was checked for normality. Non-significant interactions and factors were removed until a minimal adequate model was reached (Crawley 2013). Contrasts were assessed with the Tukey method [package lsmeans in R, Lenth (2016)].

For survival of the female spider mites, we again excluded the data from the 2nd leaf. The proportion of females surviving was analysed with a generalized linear mixed-effects model (GLMM) using the function glmer of the lme4 package in R (Bates et al. 2015), with treatment, leaf and their interaction as fixed factors and individual plant and block as random factors. The distribution of the residuals was checked for normality. All statistical analyses were performed with R Development Core Team (2017).

For the assessment of performance of aphids, a single 2- to 4-day-old apterous adult was introduced with a fine brush on leaf 3–6 of plants treated as above. Each leaf was enclosed with a bag (as above) to prevent the aphids from escaping. Four days later, all the bags were removed carefully. All adult aphids survived, except for one female, which died due to handling and was therefore excluded from the analysis. Thereafter, the number of nymphs produced by each female aphid was counted under a stereo microscope. The experiments were conducted in 2 blocks in time with 5 plants of each treatment per block. The numbers of nymphs per female per day were compared using LME as explained above with treatment, leaf and their interaction as fixed factors and individual plant and block as random factors.

Given that thrips females are able to fly and also escaped from the bags used for aphids, leaf discs were used in this experiment. Based on the results of the previous experiment, leaves 3–5 (from below) of infested and uninfested plants were used. Five leaf discs (Ø = 15 mm) were made from each leaf, avoiding areas including the midrib. Each leaf disc was placed upside down on soaked cotton wool inside a small plastic cup (Ø = 20 mm, height = 3 cm). A single 4–6 days old female thrips was released inside the cup. All cups were closed with lids with a ventilation hole covered with fine mesh (80 µm). Females were removed 3 days later and all leaf discs were kept for another 4 days until all larvae had hatched from the leaf discs. The total number of larvae that hatched from the eggs produced by each female was recorded. The experiments were conducted in 2 blocks in time and there were 8 replicates (plants) per treatment in total. Thrips that were missing or dead at the end of the experiment (around 16%) were excluded from the analysis of reproduction, but there were always surviving thrips for each leaf of each plant. We calculated the average number of thrips larvae per female of each leaf and this average was used for further analysis. As above, these data were compared using LME with leaf and treatment and their interaction as fixed factors and individual plant and block as random factors. Below, we do not report non-significant interactions. The distribution of the residuals was checked for normality. The number of thrips larvae was log (*x* + 10) transformed for the MANOVA and LME. Contrasts were assessed with the Tukey method [package lsmeans in R, Lenth (2016)].

For adult thrips, the proportions of females that survived per leaf of each plant were analysed with a GLMM as described above, with treatment, leaf and their interaction as fixed factors and individual plant and block as random factors.

### Effect of plant infestation by *M. pygmaeus* on immature herbivore development

Plants that received the same treatments (uninfested plants and plants infested by *M. pygmaeus*) as for the above experiment were used. Based on the previous experiment, leaves 3–5 of each plant were chosen for this experiment. Five leaf discs (Ø = 15 mm) were made from each leaf and placed upside down in small plastic cups on top of soaked cotton wool. Three leaves (leaves 3, 4 and 5) of five plants per treatment were used for the larval development of spider mites, and three leaves of 10 plants per treatment were used for larval development of thrips and nymphal development of aphids. A single newly hatched two-spotted spider mite larva, a newly born aphid nymph, or a first-instar thrips larva was transferred carefully to each leaf disc with a fine brush under a stereomicroscope. The cup was closed with a lid with a ventilation hole covered with fine mesh (80 µm). Juvenile survival and their developmental stages were checked under the stereomicroscope and recorded on a daily basis until the adult stage for spider mites and aphids. Thrips larvae largely stop feeding when they become prepupae; we therefore, analysed the development and survival from larva to prepupa. Discs were replaced by new ones that had received the same treatments every 3 days. Because five spider mite larvae and seven thrips larvae died due to handling, some of the averages per leaf were based four individuals instead of five. Survival of immatures per leaf was compared with a GLMM as above. The developmental times were log (*x* + 0.1) transformed and were analysed with LME following the same procedure as explained above.

### Phytohormone accumulation

To test whether plant feeding of *M. pygmaeus* induced plant defences, phytohormones were quantified. Sweet pepper plants were treated for 4 days as above, six plants with *M. pygmaeus* inside the bags, the other six plants with only the bags as control. Leaves 2–6 of each plant were frozen in liquid nitrogen and stored at − 80 °C. The metabolites OPDA, JA, JA-Ile, SA and ABA were analysed with liquid chromatography–mass spectrometry/mass spectrometry (LC–MS/MS) using the procedure of Alba et al. (2015) with some minor modifications. In short, c. 200 mg of frozen leaf material was homogenized (Precellys 24, Bertin Technologies, Aix-en-Provence, France) in 1 ml of ethyl acetate solution with 100 ng/ml of the internal standards D6-SA, D5-JA and D6-ABA (C/D/N Isotopes Inc, Canada). Samples were centrifuged at 15,000 rpm for 20 min at 4 °C and the ethyl acetate phase was transferred to new tubes. Pellets were washed with 0.5 ml of ethyl acetate without internal standards. Subsequently, the supernatants were combined with the previous extraction after centrifugation (20 min at 4 °C at 15,000 rpm). Samples were dried on a vacuum concentrator (CentriVap Centrifugal Concentrator, Labconco, Kansas City, MO, USA) at room temperature. The residue was re-suspended in 0.25 ml of 70% methanol (v/v). Samples were transferred to glass tubes and then analysed with LC–MS/MS. To calculate the amount of each compound, a serial dilution of pure standards of OPDA, JA, JA-Ile, SA, SA and traumatic acid was included. Measurements were conducted on a liquid chromatography tandem mass spectrometry system (Varian 320-MS LC/MS, Agilent Technologies, Amstelveen, the Netherlands). Twenty microlitre of each sample was injected into a Pursuit XRs 5 column (C18; 50 × 2.0 mm, Agilent Technologies, Amstelveen, The Netherlands). The mobile phase comprised solvent A (0.05% formic acid in water; Sigma-Aldrich, Zwijndrecht, the Netherlands) and solvent B (0.05% formic acid in methanol; Sigma-Aldrich). The program was set as follows: 95% solvent A for 1 min 30 s, followed by 6 min in which solvent B increased until 98% (0.2 ml min^−1^) which continued for 5 min with the same flow rate, subsequently returning to 95% solvent A for 1 min until the end of the run. Metabolites were detected using the negative electrospray ionization mode. The mother ions, daughter ions and collision energies used in these analyses are listed in Supplementary Table S1. For all oxylipins, we used D5-JA to calculate the recovery rate, and their *in planta* concentrations were subsequently quantified using the external standard series. For SA and ABA we used D6-SA and D6-ABA, respectively, to calculate the recovery rate and they were quantified using the external standard. Values were expressed as ng per gram fresh leaf material.

First, we carried out a multivariate analysis of variance (MANOVA) to check the overall effects of treatments on the concentrations of all plant hormones. Because this MANOVA showed a significant effect, we proceeded with an analysis of the individual hormones. The hormone concentrations in different leaves from each plant in each treatment were compared with LMEs with treatment, leaf and their interactions as fixed factors and individual plant as a random factor. The distribution of the residuals was checked for normality. The concentrations of OPDA and JA-Ile were square root transformed and the JA log (*x* + 1) transformed. Non-significant interactions and factors were removed until a minimal adequate model was reached (Crawley 2013).

### Vascular connectivity of different leaves of sweet pepper plants

Rhodamine-B (Sigma-Aldrich, St Louis, MO, USA) was used to track the vascular connectivity of different leaves to leaf four of sweet pepper plants following the protocol described in Orians et al. (2000) (Supp Mat Methods). Briefly, leaf tissue of leaf four of six plants was removed, and the main vein and part of the petiole was inserted in a 15 ml polypropylene tube (Greiner Bio-One GmbH, Germany) filled with a solution of Rhodamine-B 0.1% (w/v). After 4 h, 24 h, 48 h, leaves 3, 5 and 6 were excised and the distribution of Rhodamine-B in each leaf was tracked using an UV transilluminator (Syngene, UK) with an exposure time of 1.84 s for all pictures.

## Results

### Effect of plant infestation by *M. pygmaeus* on herbivore reproduction

**Fig. 1:**
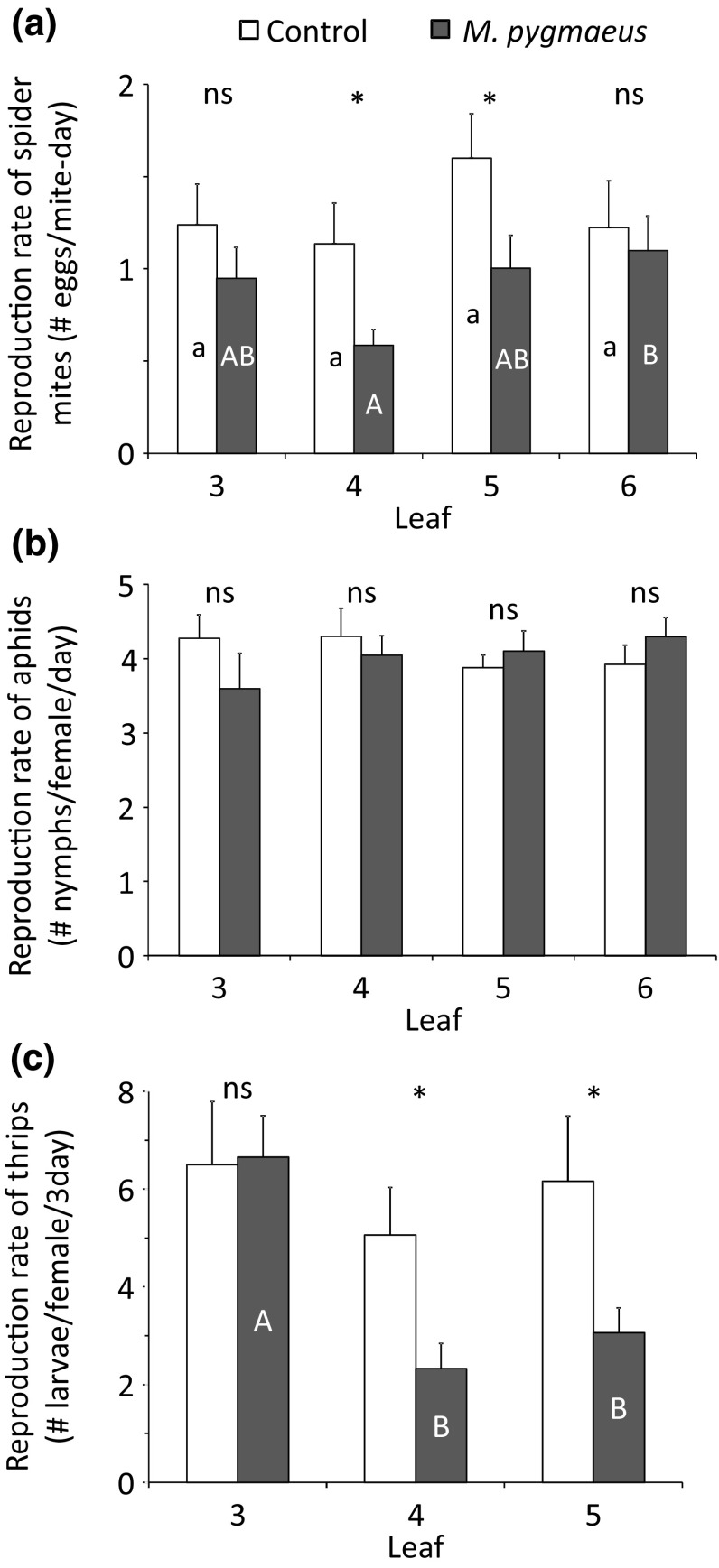
Average reproduction rate (+ SE) of *T. urticae* (**a**; *n* = 15 plants), *M. persicae* (**b**; *n* = 10) and *F. occidentalis* (**c**; *n* = 8) when feeding on leaves from plants previously exposed to *M. pygmaeus* for 4 days (black bars) and uninfested plants (control, white bars). Significant differences between corresponding leaves from infested and uninfested plants are indicated by asterisks (contrasts after LME, *P < 0.05). Different letters inside bars indicate significant differences among different leaves of uninfested plants (small letters) and of infested plants (capital letters) (contrasts after LME: *P* < 0.05)

Overall, the oviposition rate of *T. urticae* on *M. pygmaeus*-infested plants was lower than on the uninfested plants (Fig. 1a, LME: *χ* ^2^ = 5.83, *df* = 1, *P* = 0.016), and it varied among leaves (Fig. 1a, LME: *χ* ^2^ = 10.9, *df* = 3, *P* = 0.012). Female *T. urticae* feeding on the *M. pygmaeus*-damaged leaf (leaf 4) and the 5th leaf of treated plants laid significantly fewer eggs than females on corresponding leaves of uninfested plants (Fig. 1a, contrasts with glht function of package lsmeans). The same trend was observed on the 3rd and 6th leaves.

Female *M. persicae* produced similar numbers of nymphs on uninfested and treated plants (Fig. 1b, LME: *χ* ^2^ = 0.12, *df* = 1, *P* = 0.73), and numbers of nymphs did not differ among leaves (Fig. 1b, LME: *χ* ^2^ = 0.87, *df* = 3, *P* = 0.83).

Overall, lower numbers of *F. occidentalis* larvae were found on treated plants than on uninfested plants (Fig. 1c, LME: *χ* ^2^ = 4.52, *df* = 1, *P* = 0.034). Lower numbers of larvae were found on the leaf infested by *M. pygmaeus* (leaf 4) and on leaf 5 than on the corresponding leaves of uninfested plants (Fig. 1c, contrast as above). The numbers of larvae differed among leaves (Fig. 1c, LME: *χ* ^2^ = 10.9, *df* = 2, *P* = 0.004).

**Fig. 2:**
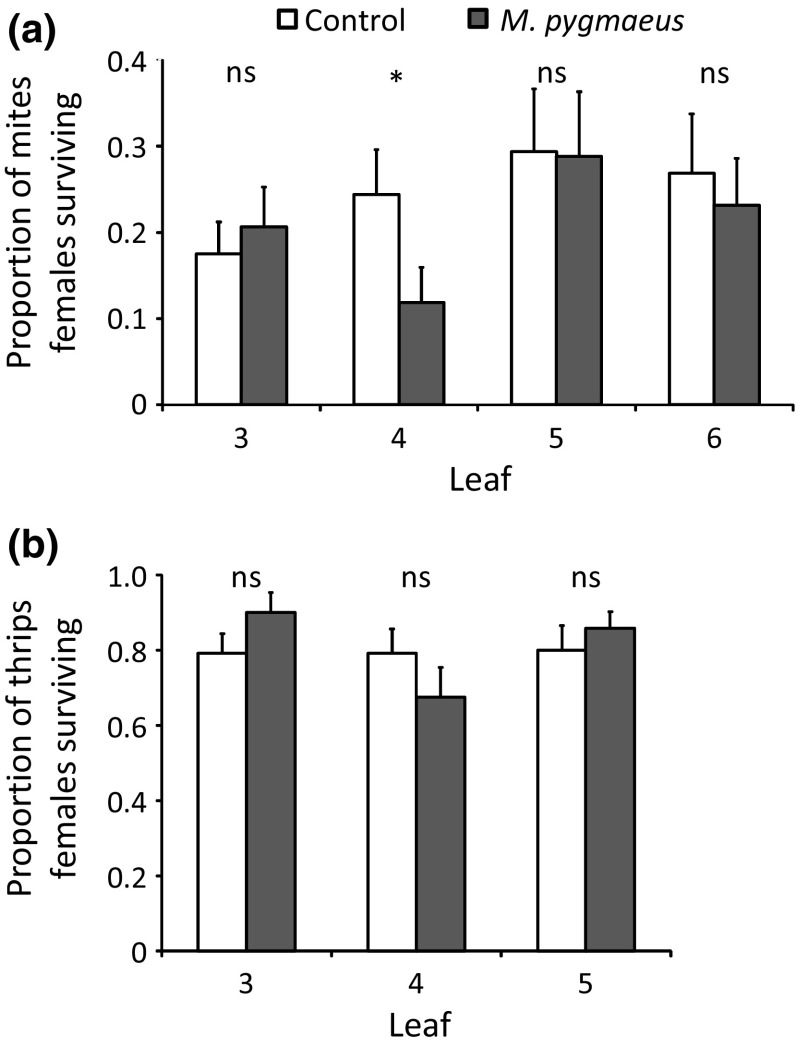
Average proportions (+ SE) of **a** *T. urticae* females surviving on leaves of plants previously exposed to *M. pygmaeus* (black bars) and leaves of uninfested plants (control, white bars) on day 4 (*n* = 15 plants), and **b** average proportions (+ SE) of *F. occidentalis* females surviving on leaves of treated and uninfested plants after 3 days (*n* = 8). Significant differences between infested and uninfested leaves are indicated by asterisks (contrasts after LME, *P < 0.05)

The survival of adult *T. urticae* females was differentially affected by feeding of *M. pygmaeus* on different leaves (Fig. 2a, GLMM, interaction between treatment and leaf: *χ* ^2^ = 7.82, *df* = 3, *P* = 0.0498). Survival on the leaf previously exposed to *M. pygmaeus* (leaf 4) was only half as high as on the corresponding leaf of uninfested plants, but survival on the other leaves did not differ significantly between treatments (Fig. 2a). All aphid females survived the entire experiment. The survival of adult *F. occidentalis* females did not differ between uninfested and *M. pygmaeus*-infested plants (Fig. 2b, GLMM: *χ* ^2^ = 0.08, *df* = 1, *P* = 0.78), or among different leaves per treatment (GLMM: *χ* ^2^ = 2.36, *df* = 2, *P* = 0.31).

### Effect of plant infestation by *M. pygmaeus* on immature herbivore development

**Fig. 3:**
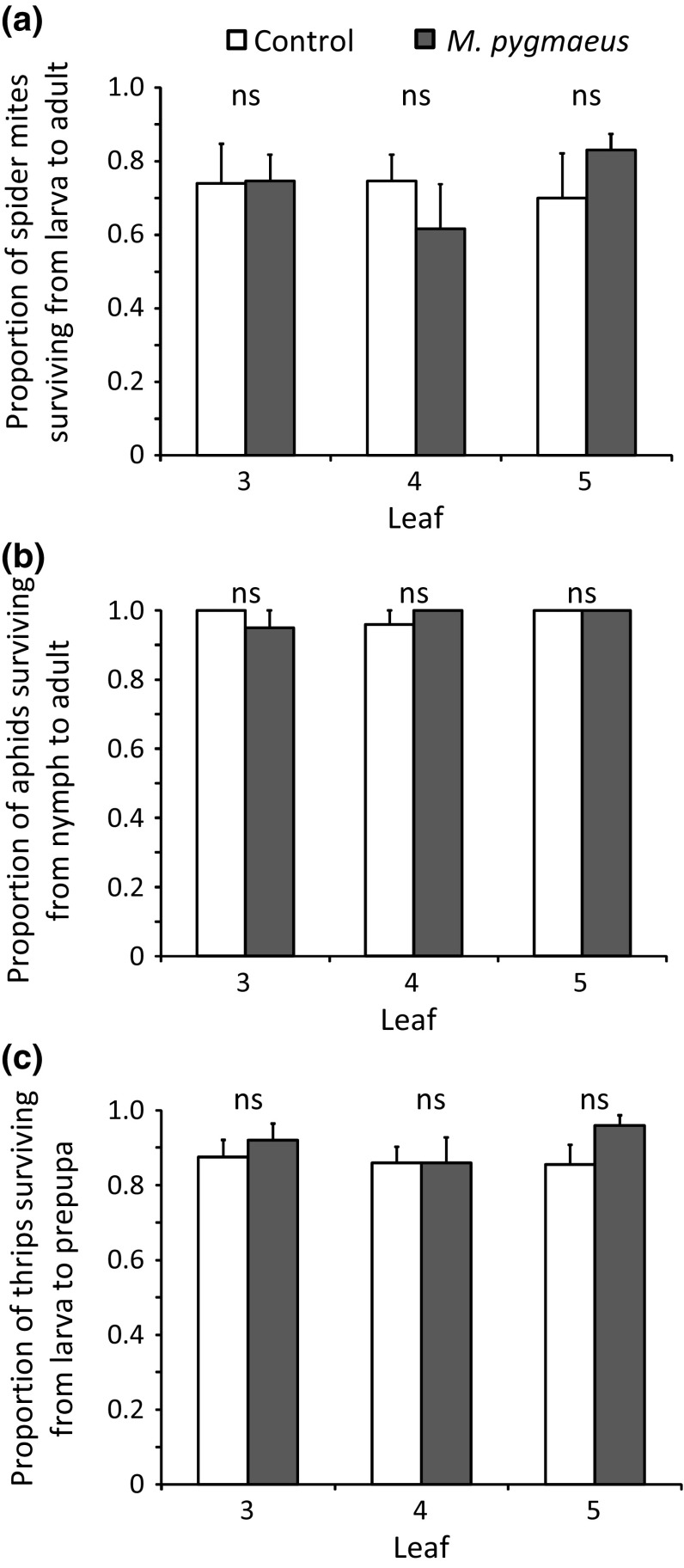
Average proportions (+ SE) of immature *T. urticae* (**a**; *n* = 5 plants) and *M. persicae* (**b**; *n* = 10) surviving to adult, and of immature *F. occidentalis* (**c**; *n* = 10) surviving to prepupa when feeding on leaves from plants previously exposed to *M. pygmaeus* for 4 days (black bars) and uninfested plants (control, white bars)

**Fig. 4:**
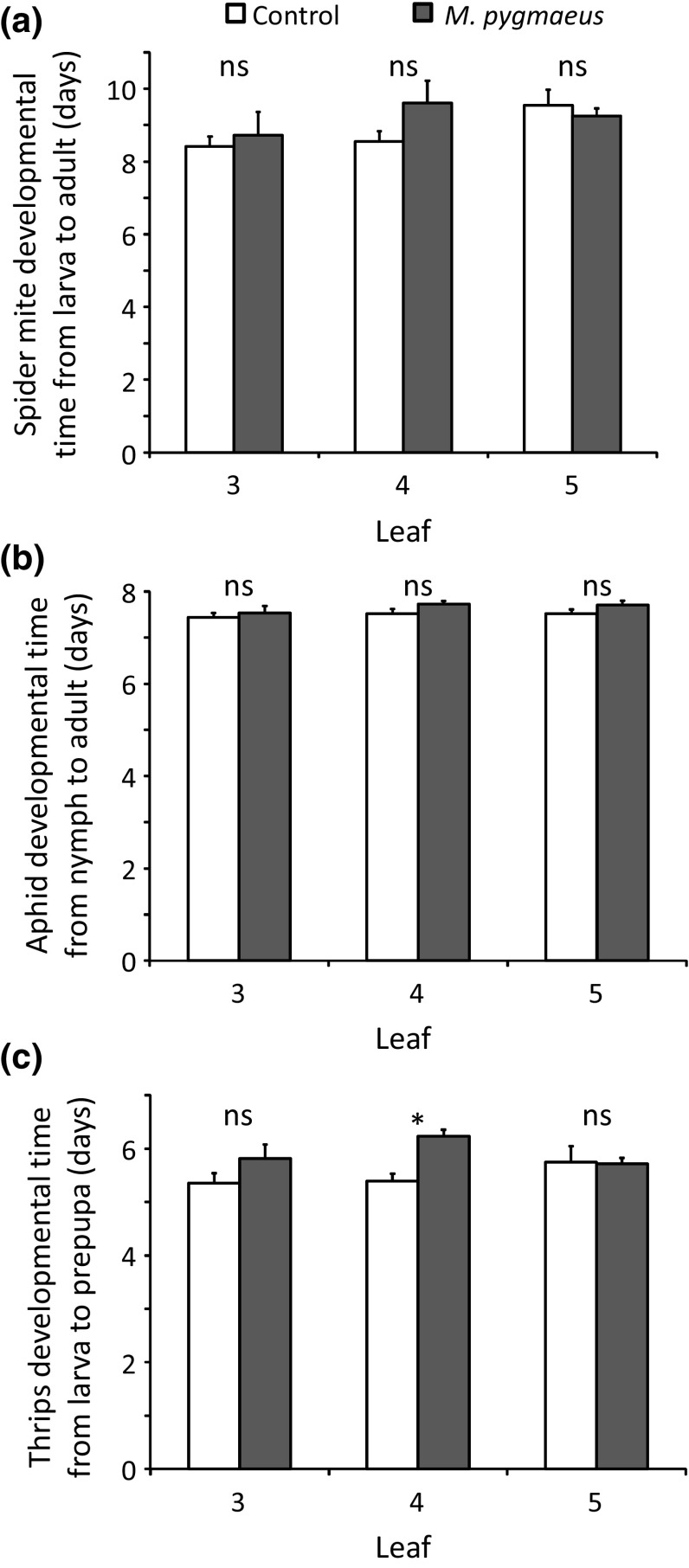
Average developmental time (+ SE) of immature *T. urticae* (**a**; *n* = 5 plants) and *M. persicae* (**b**; *n* = 10) to adult, and of *F. occidentalis* (**c**; *n* = 10) to prepupa when feeding on leaves from plants previously exposed to *M. pygmaeus* for 4 days (black bars) and uninfested plants (control, white bars). Significant differences between corresponding leaves from infested and uninfested plants are indicated by asterisks (contrasts after LME, *P < 0.05)

No significant effect of treatment or leaf was found on the survival of *T. urticae* from larva to adult (Fig. 3a, GLMM: treatment: *χ* ^2^ = 0.0008, *df* = 1, *P* = 0.98; leaf: *χ* ^2^ = 1.32, *df* = 2, *P* = 0.52). Furthermore, the developmental times of spider mite immatures feeding on uninfested and treated plants did not differ significantly (Fig. 4a, LME: *χ* ^2^ = 0.86, *df* = 1, *P* = 0.35). Developmental times of immatures feeding on different leaves of the same treatment also did not differ significantly (Fig. 4a, LME: *χ* ^2^ = 4.04, *df* = 2, *P* = 0.13).

For aphid survival, no significant effect of treatment or of leaf was found (Fig. 3b, GLMM: treatment: *χ* ^2^ = 0.82, *df* = 1, *P* = 0.78; leaf: *χ* ^2^ = 2.13, *df* = 2, *P* = 0.34). Nymphs feeding on uninfested and treated plants required similar times to develop into adults (Fig. 4b, LME: *χ* ^2^ = 2.95, *df* = 1, *P* = 0.086). The developmental period did not differ significantly on different leaves of plants of the same treatment (Fig. 4b, LME: *χ* ^2^ = 2.48, *df* = 2, *P* = 0.29).

The survival of thrips larva to prepupa was not affected by the plant treatment or the plant leaf (Fig. 3c, GLMM, treatment: *χ* ^2^ = 1.61, *df* = 1, *P* = 0.20; leaf: *χ* ^2^ = 1.24, *df* = 2, *P* = 0.54). A significant effect of the interaction between treatment and leaf was found on the developmental time from larva to prepupa (Fig. 4c, LME: *χ* ^2^ = 8.93, *df* = 2, *P* = 0.0115). Larvae feeding on treated leaves (leaf 4) required a longer time to develop into prepupae than those feeding on the corresponding leaves of uninfested plants (Fig. 4c).

### Phytohormone accumulation

Overall, there was a significant effect of the interaction between treatment and leaf on the concentrations of hormones accumulated (MANOVA: treatment: leaf: *F*_4, 24_ = 2.31, *P* = 0.009). We, therefore, analysed each plant hormone separately.

**Fig. 5:**
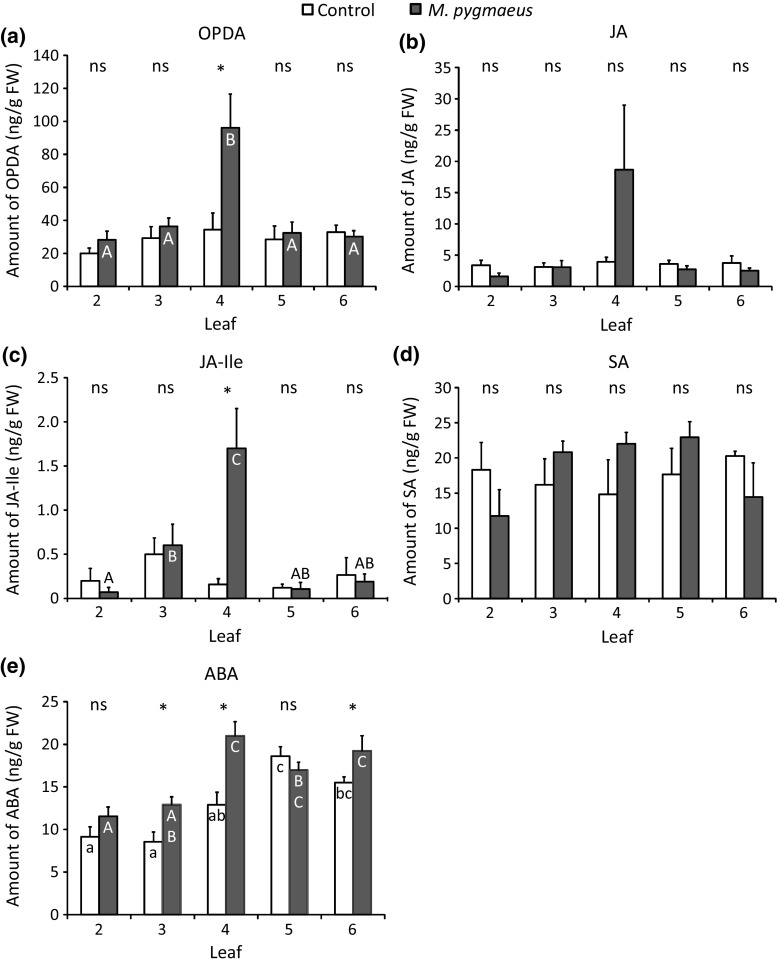
Average concentrations of plant hormones (in ng/g fresh weight), of OPDA (**a**), JA (**b**), JA-Ile (**c**), SA (**d**) and ABA (**e**) (mean + SE; *n* = 6 plants) in different leaves of plants previously exposed to *M. pygmaeus* for 4 days (black bars) and uninfested plants (control, white bars). Significant differences between corresponding leaves from infested and uninfested plants are indicated by asterisks (contrasts after LME, *P < 0.05). Different letters inside the bars indicate significant differences among different leaves of uninfested plants (small letters) and of infested plants (capital letters, contrasts after LME: *P* < 0.05)

Feeding by *M. pygmaeus* resulted in significantly higher concentrations of OPDA in the attacked leaf (leaf 4) than in the 4th leaf of the uninfested plants and than in the other leaves of the treated plants (Fig. 5a). This resulted in a significant interaction between treatment and leaf (Fig. 5a, LME: *χ* ^2^ = 11.83, *df* = 4, *P* = 0.019). Similar concentrations of OPDA were produced in all leaves of uninfested plants.

The concentrations of JA in leaves of treated and uninfested plants did not differ significantly (Fig. 5b, LME: *χ* ^2^ = 0.30, *df* = 1, *P* = 0.58), and different leaves of plants with the same treatment produced similar concentrations of JA (Fig. 5b, LME: *χ* ^2^ = 8.49, *df* = 4, *P* = 0.075). The concentration of JA in the attacked leaf (leaf 4) was higher than that in the corresponding leaf of the uninfested plants, but the difference was not statistically significant due to high variation in the JA concentration in the attacked leaves (Fig. 5b).

Leaves attacked by the omnivore (leaf 4) contained a significantly higher concentration of JA-Ile than the corresponding leaf of the uninfested plants and the other leaves of the treated plants (Fig. 5c). This resulted in a significant interaction between treatment and leaf (Fig. 5c, LME: *χ* ^2^ = 21.51, *df* = 4, *P* = 0.0003). JA-Ile concentrations in the other leaves of infested plants did not differ significantly from the corresponding leaves of uninfested plants (Fig. 5c). Different leaves of uninfested plants accumulated similar amounts of JA-Ile (Fig. 5c).

There was also a significant effect of the interaction between treatment and leaf for the amount of SA in different leaves (LME: *χ* ^2^ = 10, *df* = 4, *P* = 0.040), but there was no significant effect of treatment per leaf (Fig. 5d). Within each treatment, there was no significant difference in the concentrations of SA among leaves (Fig. 5d, contrasts as above).

Feeding by *M. pygmaeus* also resulted in significantly higher concentrations of ABA in the attacked leaf (leaf 4) and in the uninfested leaves 3 and 6 than in the other leaves of the treated plants and than in the corresponding leaves of the uninfested plants (Fig. 5e, LME, interaction between treatment and leaf: *χ* ^2^ = 16.76, *df* = 4, *P* = 0.002). Different leaves within the same treatment accumulated different amounts of ABA (Fig. 5e).

### Vascular connectivity of different leaves of sweet pepper plants

After 4 h, Rhodamine-B was observed in half of each leaf 5 and 6, but not in leaf 3 (Supp Mat Figure S1). Subsequently, the Rhodamine-B also accumulated in the other halves of leaf 5 and 6 after 24 h (Supp Mat Figure S1). After 48 h, it was visible in leaf 3 and both sides of the leaf 5 and 6 (Supp Mat Figure S1).

## Discussion

We show that the feeding of *M. pygmaeus* on sweet pepper plants lowers the performance of two of the three herbivore species feeding on the same plants through induced defences. The reproduction of *T. urticae* and *F. occidentalis* on *M. pygmaeus*-infested plants was significantly lower than on uninfested plants, not only on the leaves that had been exposed to *M. pygmaeus*, but also on other leaves of the same plants, showing that the effect was systemic. Furthermore, *F. occidentalis* larvae developed slower on leaves previously exposed to *M. pygmaeus* than on uninfested leaves. In contrast, there was no effect on the reproduction of female *M. persicae*. The developmental rate and juvenile survival of *T. urticae* and *M. persicae* and larval survival of *F. occidentalis* were not affected by the previous infestation of *M. pygmaeus*.

Similarly, Pappas et al. (2015) have shown that the performance of *T. urticae* was lower on local and systemic leaves of tomato plants that were previously exposed to *M. pygmaeus* than on leaves of uninfested plants, but they found no effect on the greenhouse whitefly. However, these authors found lower survival and oviposition of spider mites, but it is unclear whether the lower oviposition was mainly caused by decreased survival of the ovipositing female, by surviving females producing fewer eggs, or by both. Here, we corrected for mortality of the adult females and show that both the survival and oviposition rate were negatively affected by previous exposure of plants to *M. pygmaeus*. Oviposition rates of spider mites on uninfested plants were relatively low (1–1.5 eggs per day), confirming that sweet pepper is a less suitable host plant for this herbivore (van den Boom et al. 2003). Another study showed that the predatory bug *O. laevigatus* also increased tomato resistance against the thrips *F. occidentalis* (De Puysseleyr et al. 2011). Thus, omnivorous predators can decrease the performance of herbivores sharing the same plants both directly, through predation, as well as indirectly through induced plant defences.

The number of spider mite females on the *M. pygmaeus*-infested plants decreased during the experiments compared to those on the uninfested plants. Because there were no predators present, the females either escaped or died because of poor plant quality. Further experiments are needed to confirm whether spider mites disperse more from plants previously exposed to *M. pygmaeus*. Notice that reproduction was corrected for this dispersal and mortality (see “Materials and methods”). Because thrips and aphid reproduction was tested with leaf discs and in bags, respectively, they were prevented from escaping.

Besides reacting to reduced plant quality due to induction of defences, the herbivores may have responded to the cues left behind by the omnivore on the exposed leaves, as was found for several prey in response to predator cues (Kats and Dill 1998). Possibly the presence of omnivore faeces or other products may have affected the behaviour of the herbivores. It is also known that egg deposition by herbivores (Hilker and Meiners 2006) or even herbivore walking (Bown et al. 2002; Peiffer et al. 2009) can induce changes in plants. Perhaps plants respond in a similar manner to omnivores and this may have contributed to modulation of the plant’s local and systemic defence responses. However, the reproduction of spider mites and thrips was also reduced on unexposed leaves of exposed plants, suggesting that plant quality affected the performance of the herbivores.

In contrast to reproduction, the juvenile survival and developmental period of *T. urticae* and the juvenile survival of *F. occidentalis* were not affected by the infestation of *M. pygmaeu*s, but the juvenile developmental time of *F. occidentalis* was delayed on leaves previously exposed to *M. pygmaeu*s. This may point at a difference in response of juveniles versus adults to cues associated with previous exposure of the plants. Possibly, adult females refrain from reproducing in the presence of such cues. This could be caused by egg retention by the females (Montserrat et al. 2007), or because the females attempted to escape, thus spending less time and energy on reproduction. The observation that many spider mites did escape or die partly confirms this, but even females that did not escape reproduced less. Although thrips and aphid females were confined, this did not prevent them from attempting to escape and feeding and reproducing less as a consequence. Another explanation would be that juvenile spider mites and thrips feed much less than adult females, hence, suffer less from the decreased plant quality. Furthermore, we used leaf discs for the experiments on larval development experiments instead of intact plants for practical reasons, which may also have affected the results. Although both methods are amply used in the literature, the effects of induced plant defences may differ between these two approaches. Elsewhere (Dias et al., in prep.) we address this issue, showing that the reproduction rates of spider mites on leaf discs and on entire plants showed the same trend. Therefore, it is likely that the effect of infestation by *M. pygmaeus* on thrips reproduction will be comparable on leaves from intact plants.

In the current experiments, five pairs of *M. pygmaeus* were released on the 4th leaf of each plant, and no leaf damage by *M. pygmaeus* was observed during the experiments, yet these low numbers were sufficient to activate plant defences. In our experiments, *M. pygmaeus* were released on one leaf, however, in practice, they are free to disperse to other plant parts, and hence more leaves may become exposed to these omnivores, resulting in larger overall effects on herbivore performance. However, pest individuals may actively avoid feeding and reproducing on leaves that were previously exposed to *M. pygmaeus*. This will be the subject of further research.

Oviposition of female *M. pygmaeus* and plant feeding of nymphs can also induce plant defences (Pappas et al. 2015). In this study, no prey or food was supplied to *M. pygmaeus.* An earlier study showed that female *M. pygmaeus* did not oviposit on pepper plants in the absence of prey (Perdikis and Lykouressis 2004), and indeed, no nymphs were observed on the plants during our experiments. Thus, the effect on the performance of herbivores was mainly due to the response induced by the feeding of *M. pygmaeus* and not due to the response to oviposition or to the presence of nymphs.

The three herbivores species used in our study have different feeding modes and induce and are sensitive to different plant defences. Aphids are sensitive to the JA-related defences, but they mainly induce SA-related defences, which suppress JA-related defences (Omer et al. 2001; Zarate et al. 2007; Walling 2008; Puthoff et al. 2010). Thrips are sensitive to JA-related defences and spider mites are sensitive to both JA- and SA-related defences. Our observation that the performance of spider mites and thrips were affected by plant feeding by *M. pygmaeus* and that of the aphids not, suggests that the omnivore mainly induces JA-related defences.

To further confirm whether plant defences were involved in the decrease of performance of some of the herbivores tested here, we quantified the plant hormones accumulated in leaves of uninfested plants and *M. pygmaeus*-infested plants. We found that the concentrations of the plant hormones OPDA and JA-Ile in the exposed leaves was significantly higher than in the corresponding leaves of uninfested plants, but no such effect was found for the uninfested leaves of the exposed plants. The accumulation of JA showed the same trend as that of JA-Ile, but this was not significant. Concentrations of JA and JA-Ile in induced plants were comparable to those found in induced tomato plants (Alba et al. 2015; Schimmel et al. 2017). The amount of SA in the 3rd, 4th, 5th leaves of treated plants was not significantly higher than in corresponding leaves of the uninfested plants. ABA levels were significantly higher in leaves exposed to *M. pygmaeus* and in some of the other leaves of the same plants than in the respective leaves of the uninfested plants. Similarly, Pérez-Hedo et al. (2015b) found that the JA signalling pathway was activated and the amount of ABA was higher in tomato leaves attacked by the omnivore *N. tenuis*, but the SA pathway was not activated. Attack by *M. pygmaeus* only activated the JA signalling pathway in tomato leaves, not the ABA pathway (Pérez-Hedo et al. 2015a). It is likely that plants of different species respond differently to omnivore damage. It is known that ABA plays an important role in response to abiotic stress and its role in biotic stress is becoming evident in *Arabidopsis* (Mauch-Mani and Mauch 2005; Asselbergh et al. 2008; Pieterse et al. 2009). In *Arabidopsis*, ABA enhances the JA-related response (Anderson et al. 2004) and antagonizes SA-dependent responses (Mohr and Cahill 2007). All these results suggest that several of the plant hormones investigated here are involved in the local defence induced by the omnivore, but are not decisive in the systemic defences experienced by the herbivores. Perhaps other, unidentified metabolites (e.g. OPC4; Gasperini et al. 2015) are involved in the systemic effects. Alternatively, plant defences may have been primed in undamaged distal leaves of *M. pygmaeus*-infested plants, and JA-regulated defences may not have been activated by *M. pygmaeus* feeding, but could have been rapidly activated upon subsequent attacks by the herbivores, which would explain the decreased performance of herbivores on these leaves. The systemic response by ABA suggests that it may play a role in modulating JA-defences induced by omnivorous mirid bugs. Indeed, ABA plays an important role in herbivore-induced defences by activating primed JA-regulated defences upon secondary herbivore attack (Vos et al. 2013).

Concluding, our results suggest that plant feeding by the omnivorous predator *M. pygmaeus* induces the JA-related defensive pathway in sweet pepper plants, and this coincides with reduced performance of the herbivore species *T. urticae* and *F. occidentalis*. The phytohormones accumulated in the *M. pygmaeus*-infested plants confirm that mainly the JA-related pathway was induced. Perhaps aphids can decrease these defences by inducing SA-related defences. Pappas et al. (2015) found no effect of previous exposure of tomato plants on the performance of whitefly, which is also considered to be able to suppress induced JA-related defences (Zarate et al. 2007; Walling 2008). Together, this suggests that spider mites and thrips are more sensitive to the defences induced by *M. pygmaeus* than are whiteflies and aphids, perhaps because the latter can decrease these defences through cross-talk with the SA-related defences they induce.

*Macrolophus pygmaeus* attacked the 4th leaf of the plants in our experiment. This leaf has higher vascular connectivity with leaves 5 and 6 than with leaf 3 (Supp Mat Figure S1). However, the dye used in this experiment was also detected in leaf 3 after 48 h (Supp Mat Figure S1). Because our plants were treated with *M. pygmaeus* for 4 days, defence-related compounds were likely transferred to all untreated leaves during this time, enabling systemic effects on herbivores feeding on the tested leaves. Although the increased concentrations of OPDA, JA and JA-Ile point at a local, non-systemic effect, the concentrations of ABA point at a systemic effect. Perhaps longer exposure of plants to the omnivores, or exposure to higher densities, would result in more pronounced differences in phytohormone concentrations in distal leaves. Alternatively, the systemic response may have been primed rather than induced (Conrath et al. 2015) which implies that systemic effects could only have been seen when comparing primed and unprimed leaves infested with herbivores. This will be the subject of further research.

Overall, we conclude that plant feeding by the omnivorous predator *M. pygmaeus* can decrease the performance of herbivores feeding on the same plants through the induction of defences. Possibly plants simply responded to the feeding of the omnivores similar to their response to herbivores. However, plants are known to respond differently to different herbivore species, suggesting that there is specificity in their response (Turlings et al. 1998; De Moraes et al. 1998; Alba et al. 2015). Hence, plants can adapt their defensive response to the agent causing the plant damage on an evolutionary time scale, so induction of defences by *M. pygmaeus* may not be a simple side effect of plant damage. A remaining question is then why plants would mount direct defences when they are already defended by omnivorous predators. Possibly, plants affect the omnivore’s diet through induced defences, thus manipulating them to feed more on herbivores and less on the plant tissue in which defences are induced. It is known that omnivores may change their diet according to plant quality (Agrawal et al. 1999; Janssen et al. 2003), and that a decrease in plant quality may increase the predation rate of omnivores (Eubanks and Denno 2000). Further research is, therefore, needed to investigate the diet choice of *M. pygmaeus* on induced and uninduced plants. Another possibility is that in nature, the presence of omnivorous predators is strongly correlated with the presence of herbivores, and thus, plant defences are likely to be induced anyway, so further induction by omnivores has little effect on plant defences and plant fitness. Clearly, further research is needed to investigate the ecological role of omnivorous predators in plant–herbivore–predator interactions.

## Electronic supplementary material

Below is the link to the electronic supplementary material.

Supplementary material 1 (DOCX 1404 kb)
