# Correction to: Phytophagy of omnivorous predator *Macrolophus pygmaeus* affects performance of herbivores through induced plant defences

**DOI:** 10.1007/s00442-017-4015-0

**Published:** 2017-11-27

**Authors:** Nina Xiaoning Zhang, Gerben J. Messelink, Juan M. Alba, Robert. C. Schuurink, Merijn R. Kant, Arne Janssen

**Affiliations:** 1 IBED, Population Biology, University of Amsterdam, Science Park 904, 1098 XH Amsterdam, The Netherlands; 2 Wageningen UR Greenhouse Horticulture, PO Box 20, 2265 ZG Bleiswijk, The Netherlands; 3 Department of Plant Physiology, Swammerdam Institute for Life Sciences, University of Amsterdam, P.O. Box 94215, 1090 GE Amsterdam, The Netherlands

## Correction to: Oecologia 10.1007/s00442-017-4000-7

Unfortunately, the citation of one of the papers was published erroneously in the original version and corrected here by this Erratum. The original article was corrected.

In the discussion section Paragraph six should read as:

In the current experiments, five pairs of *M. pygmaeus* were released on the 4th leaf of each plant, and no leaf damage by *M. pygmaeus* was observed during the experiments, yet these low numbers were sufficient to activate plant defences. In our experiments, *M. pygmaeus* were released on one leaf, however, in practice, they are free to disperse to other plant parts, and hence more leaves may become exposed to these omnivores, resulting in larger overall effects on herbivore performance. However, pest individuals may actively avoid feeding and reproducing on leaves that were previously exposed to *M. pygmaeus*. This will be the subject of further research.

