# Supplementary material for: Phytophagy of omnivorous predator Macrolophus pygmaeus affects performance of herbivores through induced plant defences
Source: Oecologia. 2017 Nov 9;186(1):101–13. doi: 10.1007/s00442-017-4000-7 (PMC5756286; doi:10.1007/s00442-017-4000-7)
Supplement: Supplementary file 1 — Supplementary material 1 (DOCX 1404 kb) [file 442_2017_4000_MOESM1_ESM.docx]

Supplementary Materials for

Phytophagy of omnivorous predator *Macrolophus pygmaeus* affects performance of herbivores through induced plant defences

Nina Xiaoning Zhang, Gerben J. Messelink, Juan M. Alba, Robert. C. Schuurink, Merijn R. Kant, Arne Janssen*

*Corresponding author. Email: arne.janssen@uva.nl

**Supplementary Data Description**

**Supplementary Results** Spread of Rhodamine-B through sweet pepper plants

**Supplementary Table S1** Parameters used for detection of phytohormones and related compounds with LC-MS/MS

**Supplementary Figure S1** Presence of Rhodamine-B in sweet pepper leaves 3, 5 and 6 after 4h, 24h, and 48h.

**Supplementary References**

**Supplementary Results**

**Spread of Rhodamine-B through sweet pepper plants**

After 4h, Rhodamine-B was observed in half of leaf 5 and 6, but not in leaf 3 (Figure S1), suggesting that half of the leaves 5 and 6 were connected with leaf 4 through vascular bundles, but leaf 3 was not directly connected. Subsequently, the Rhodamine-B also accumulated in the other halves of leaf 5 and 6 after 24 h (Figure S1). After 48h, it was visible in leaf 3 and both sides of leaves 5 and 6.

**Table S1** Parameters used for detection of phytohormones and related compounds with LC-MS/MS

| Compound | Capillary  CID^1^  (V) | Molecular ion [M-H] (*m/z*) | Fragment ion  (*m/z*) | CE^2^  (V) | Reference |
| --- | --- | --- | --- | --- | --- |
| OPDA | -35 | 291 | 165 | 18 | (Koo et al. 2009) |
| JA | -35 | 209 | 59 | 12 | (Wu et al. 2007) |
| D_5_-JA (IS) | -35 | 213 | 61 | 12 | (Alba et al. 2015) |
| JA-Ile | -35 | 322 | 130 | 19 | (Wu et al. 2007) |
| SA | -35 | 137 | 93 | 15 | (Wu et al. 2007) |
| D_6_-SA (IS) | -35 | 141 | 97 | 15 | (Alba et al. 2015) |
| ABA | -35 | 263 | 153 | 9.0 | (Bonaventure et al. 2011) |
| D_6_-ABA(IS) | -35 | 269 | 159 | 9.0 | (Bonaventure et al. 2011) |

^1^collision-induced dissociation; ^2^collision energy; IS = internal standard.

**Fig. S1** Presence of Rhodamine-B in sweet pepper leaves 3, 5 and 6 after 4h, 24h, and 48h. Rhodamine-B was applied to the main vein and petiole of leaf 4

**Supplementary References**

Alba JM, Schimmel BCJ, Glas JJ, et al (2015) Spider mites suppress tomato defenses downstream of jasmonate and salicylate independently of hormonal crosstalk. New Phytol 205:828–840

Bonaventure G, VanDoorn A, Baldwin IT (2011) Herbivore-associated elicitors: FAC signaling and metabolism. Trends Plant Sci 16:294–299

Koo AJK, Gao X, Daniel Jones A, Howe GA (2009) A rapid wound signal activates the systemic synthesis of bioactive jasmonates in *Arabidopsis*. Plant J 59:974–986

Wu J, Hettenhausen C, Meldau S, Baldwin IT (2007) Herbivory rapidly activates MAPK signaling in attacked and unattacked leaf regions but not between leaves of *Nicotiana attenuata*. Plant Cell 19:1096–1
